# Relationships between Easily Available Biomarkers and Non-Dipper Blood Pressure Pattern in Patients with Stable Coronary Artery Disease

**DOI:** 10.3390/life13030640

**Published:** 2023-02-25

**Authors:** Andrei Drugescu, Mihai Roca, Ioana Mădălina Zota, Alexandru-Dan Costache, Maria-Magdalena Leon-Constantin, Oana Irina Gavril, Radu Sebastian Gavril, Teodor Flaviu Vasilcu, Ovidiu Mitu, Cristina Mihaela Ghiciuc, Florin Mitu

**Affiliations:** 1 Medical I Department, Faculty of Medicine, “Grigore T. Popa” University of Medicine and Pharmacy, 700115 Iasi, Romania; 2 Morpho-Functional Sciences II Department, Faculty of Medicine, “Grigore T. Popa” University of Medicine and Pharmacy, 700115 Iasi, Romania

**Keywords:** biomarkers, hypertension, non-dipper pattern, coronary artery disease

## Abstract

Introduction. Chronic inflammation plays an essential role in the pathophysiology of both arterial hypertension (HTN) and coronary artery disease (CAD), and is more pronounced in individuals with a non-dipper circadian blood pressure (BP) pattern. A non-dipping BP pattern is in turn is associated with increased cardiovascular morbi-mortality, and a higher risk of atherosclerotic events. Neutrophil to lymphocyte ratio (NLR), monocyte to lymphocyte ratio (MLR) and platelet to lymphocyte ratio (PLR) are readily available predictors of systemic inflammation and cardiovascular risk. The purpose of our study is to evaluate whether NLR, MLR and PLR can be used as cost-effective predictors of a non-dipping blood pressure pattern in hypertensive patients with stable CAD. Materials and Methods: We performed a cross-sectional retrospective analysis that included 80 patients with hypertension and stable CAD (mean age 55.51 ± 11.83 years, 71.3% male) referred to a cardiovascular rehabilitation center. All patients underwent clinical examination, 24 h ambulatory blood pressure monitoring (ABPM) and standard blood analysis. Results: Baseline demographic characteristics were similar in both groups. Patients with non-dipper pattern had significantly higher NLR (median = 2, IR (2–3), *p <* 0.001), MLR (median = 0.31, IR (0.23–0.39), *p* < 0.001) and PLR (median = 175, IR (144–215), *p* < 0.001) compared to dippers. Conclusion: Our results suggest that MLR and PLR are inexpensive and easily accessible biomarkers that predict a non-dipping pattern in hypertensive patients with stable CAD.

## 1. Introduction

The global burden of hypertension (HTN) is increasing worldwide, partly due to the current obesity pandemic and to the increased prevalence of sedentarism and unhealthy lifestyle choices. Apart from it being a disease per se, HTN is also considered a major modifiable risk factor for cardiovascular morbidity and mortality [1]. Medical progress has facilitated the diagnosis of chronic HTN and has developed a wide range of pharmacological and non-pharmacological instruments that are effective in controlling blood pressure (BP) values. However, treatment adherence and BP control are below optimal in Romania and in most of Eastern Europe, as shown by recent studies [2].

The most recent European Society of Cardiology (ESC) guidelines have revised the standard HTN diagnostic protocol. Current standard is that the patient should undergo repeated office BP measurements, as well as home-based blood pressure monitoring (HBPM) or ambulatory blood pressure monitoring (ABPM). ABPM is a readily available, noninvasive instrument that assesses circadian BP variability and characterizes the individual dipping status. Current guidelines recommend an increased use of ambulatory devices, since ABPM is a more reliable tool for HTN diagnosis and control assessment, but also for BP profile characterization. This is useful in predicting HTN-mediated cardiovascular risk and target organs damage [3]. BP values follow a circadian pattern, being higher during daytime and normally decreasing by 10–20% at nighttime [4]. Individuals who exhibit this physiological decline are known as “dippers”, while those who present a blunted decrease in nighttime systolic BP values (0–10%) are considered “non-dippers”. Approximately 25% of hypertensive patients exhibit a non-dipping BP profile, which is favored by the presence of diabetes, renal disease, autonomic neuropathies and old age [5]. BP values during the night are also more important in the assessment of cardiovascular risk, compared to daytime BP, as is decreased blood pressure variability registered on ABPM measurement [6,7,8]. Interestingly, an extreme dipping pattern (a reduction in SBP above 20% during nighttime) does not impact cardiovascular risk until the age of 70. However, above this age, an extreme dipping pattern is associated with a four-time increase in cardiovascular risk [9].

Similarly to the ESC guidelines, the most recent American College of Cardiology and American Heart Association (ACC/AHA) guidelines on hypertension also recommend an increased use of ambulatory blood pressure monitoring in order to accurately characterize circadian BP profile [10,11,12].

Current research supports the fact that HTN and coronary artery disease (CAD) both have inflammatory components [13,14,15]. Persistently elevated cytokine concentrations promote endothelial dysfunction and impaired vasodilation, explaining the association between chronic inflammation, HTN and accelerated atherosclerosis [16]. Moreover, previous studies have documented a more pronounced inflammatory response in both hypertensive and normotensive individuals that present a non-dipping BP pattern [17,18,19].

In order to further investigate the inflammatory pathways in cardiovascular diseases, one of the most facile methods is the platelet to lymphocyte ratio (PLR). Though its initial use was that of a prognostic biomarker in oncological patients [20,21], its role has since extended in the field of cardiology, in pathologies such as heart failure [22,23,24], acute coronary syndromes [25,26,27,28,29], atrial fibrillation [30], deep venous thrombosis [31] or in patients undergoing cardiovascular rehabilitation programs [32]. MLR has recently emerged as a sensitive inflammatory marker with prognostic valences in oncology [33,34], preeclampsia [35], COVID-19 [36] and CAD [37].

Apart from PLR and MLR, the neutrophil to lymphocyte ratio (NLR) is a parameter with ease of determination and use in cardiovascular diseases [38,39,40]. Two previous studies showed that elevated NLR and PLR can predict a non-dipper status in hypertensive patients. As such, we hypothesized that NLR, MLR and PLR could be a useful tool in distinguishing dipper versus non-dipper BP profile in hypertensive patients with stable CAD [41,42]. NLR, MLR and PLR, are all accessible composite ratios that combine different inflammatory parameters, which may be able to provide additional information regarding the immunological pathogenesis of BP variability [43].

## 2. Materials and Methods

We performed a retrospective cross-sectional study of all hypertensive patients with stable CAD referred for cardiac rehabilitation between January 2020 and June 2021 in the Cardiovascular Unit of the Clinical Rehabilitation Hospital (Iasi, Romania), a nationally accredited clinic specialized in phase II-III cardiovascular rehabilitation [44]. Our study sample included hypertensive patients aged 18 or older, previously diagnosed with stable CAD and who underwent ABPM upon admission. Patients with ACS during the prior 12 months, anemia, paroxysmal, persistent or permanent atrial fibrillation, moderate or severe valvulopathy, decompensated congestive heart failure, or any other severe chronic disorder except CAD were excluded from our study. Furthermore, in line with the scope of our study, patients with acute or recent (past 30 days) infections were excluded from the analysis. All patients tested negative for COVID-19 (PCR) upon hospital admission. ABPM results and demographic, clinical, and biological data were obtained from official medical records.

All patients enrolled in the study were under optimal therapy, according to current European treatment guidelines [3,45]. High blood pressure (HTN) was defined as current BP lowering therapy, resting systolic blood pressure (SBP) ≥ 140 and diastolic blood pressure (DBP) ≥ 90 mmHg, average BP/24 h ≥ 130/80 mmHg, daytime BP average ≥ 135/85 mmHg and nighttime BP average ≥ 120/70 mmHg [3]. HTN was classified in grade 1 (SBP 140–159 mmHg and/or DBP 90–99 mmHg), grade 2 (SBP 160–179 mmHg and/or DBP 100–109 mmHg) and grade 3 (SBP ≥ 180 mmHg and/or DBP ≥ 110 mmHg) according to current guidelines [3]. Stable CAD was defined as the presence of typical angina pectoris and/or positive stress test, previous coronary revascularization or history of an acute coronary syndrome more than 1 year prior to current hospital admission [45]. Obesity was defined as a body mass index (BMI) ≥30 kg/m2. Diabetes was defined as a previous diagnosis of diabetes, current antidiabetic therapy, fasting glucose ≥ 126 mg/dL obtained on two separate occasions or glycosylated hemoglobin (HbA1c) ≥ 6.5% [46].In line with hospital internal protocol, all blood samples were fasting blood samples collected in the morning upon admission, by qualified medical providers, and were processed in the same day in the hospital’s internal laboratory. Complete blood count was processed using the Pentra DF Nexus Hematology System® (Horiba Healthcare, Kyoto, Japan). Biochemistry was processed using the Transasia XL 1000 Fully Automated Biochemistry Analyzer (Transasia Bio-Medicals Ltd., Mumbai, India). We collected the following biomarkers: platelet, neutrophil, lymphocyte and monocyte count, low-density lipoprotein (LDL), high-density lipoprotein (HDL), HbA1c, erythrocyte sedimentation rate (ESR) and C reactive protein (CRP). We calculated NLR using the absolute neutrophil (N) and lymphocyte (L) values by the following formula: NLR = N/L. We calculated PLR using the absolute platelets (P) and lymphocyte (L) values, by the following formula: PLR = P/L. We calculated MLR using the absolute monocyte (M) and lymphocyte (L) values, by the following formula: MLR = M/L.

All patients underwent 24 h ambulatory BP monitoring using DMS-300 ABP (DM Software, Stateline, NV, USA). Automatic BP measurement were obtained every 30 min during daytime (07:00—23:00), and every 60 min during nighttime (23:00—07:00). Patients were instructed to remain silent and still during each automatic BP measurement. An ABPM recording was regarded valid and included in the analysis if it encompassed at least 70% successful BP recordings. Average 24 h, daytime and nighttime SBP and DBP were extracted from the ABPM report. BP dipping was computed by the following equation: (%) 100 × [(daytime SBP—nighttime SBP /daytime SBP]. A normal BP dipping index was defined as a 10–20% decrease in average nocturnal SBP compared to the average diurnal SBP. A non-dipping pattern was defined by a 0–0.9% decrease in average nocturnal SBP pattern compared to the average diurnal SBP. After assessing BP dipping index, we divided our initial study population into two subgroups: patients with a normal dipping pattern versus patients with a non-dipping pattern.

### Statistical Analysis

We analyzed the normality of distribution of continuous data using the Shapiro–Wilk test. Continuous variables with normal distribution are presented as mean ± standard deviation (SD). Non-normally distributed continuous variables are presented as median with interquartile range. Categorical variables are listed as number of cases (N) with percent frequency (%). An independent samples T-test was applied to compare continuous variables with normal distribution. A non-parametric Mann–Whitney’s U test was used to compare non-normally distributed continuous variables. A *p* value < 0.05 was considered the threshold for statistical significance. Variables with *p < 0.05* in the descriptive analysis were included in the multivariate logistic regression model, to assess the independent predictors of non-dipper pattern. The results are presented as odds ratio (OR) with 95% confidence intervals (CIs). Statistical analysis was performed in SPSS 20.0 (Statistical Package for the Social Sciences, Chicago, IL, USA).

The study received approval from the Review Board/Ethics Committee of the Clinical Rehabilitation Hospital (Iasi, Romania) (28567/21 December 2020) and of University of Medicine and Pharmacy “Gr. T. Popa” Iasi and complied with the Declaration of Helsinki. Informed consent was considered unnecessary, due to the retrospective design of this research (retrospective database analysis).

## 3. Results

Our study included 80 patients (57 males, 23 females) with a higher prevalence of grade 3 HTN (53%). Table 1 shows clinico-demographic characteristics and laboratory findings of the 80 analyzed patients and univariate analysis of the two subgroups according to dipping status. Age, the distribution of the 3 HTN grades and the prevalence of cardiometabolic comorbidities (body mass index (BMI), diabetes, LDL level, HDL level and HDL to LDL ratio) were similar among the two subgroups.

**Figure 1:**
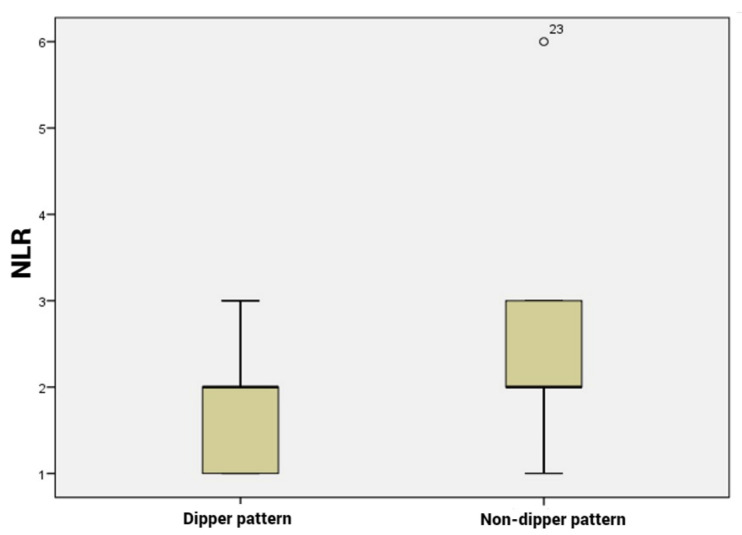
Neutrophil to lymphocyte ratio levels according dipper vs. non-dipper pattern.

**Figure 2:**
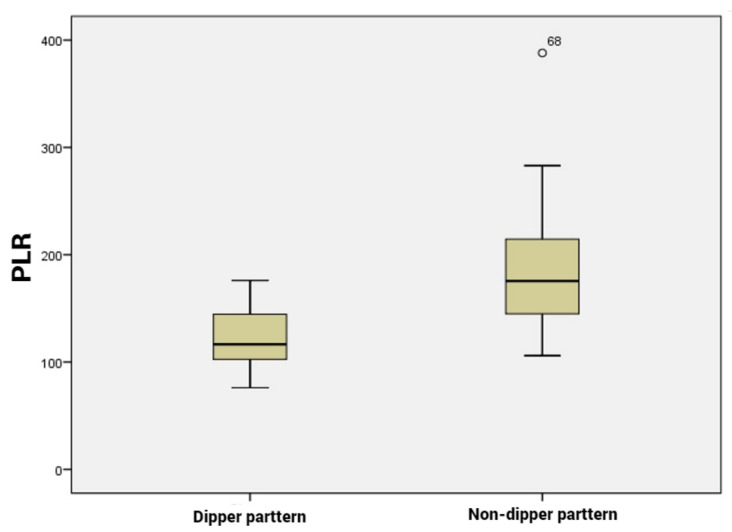
Platelet to lymphocyte ratio levels according to dipper vs. non-dipper pattern.

**Figure 3:**
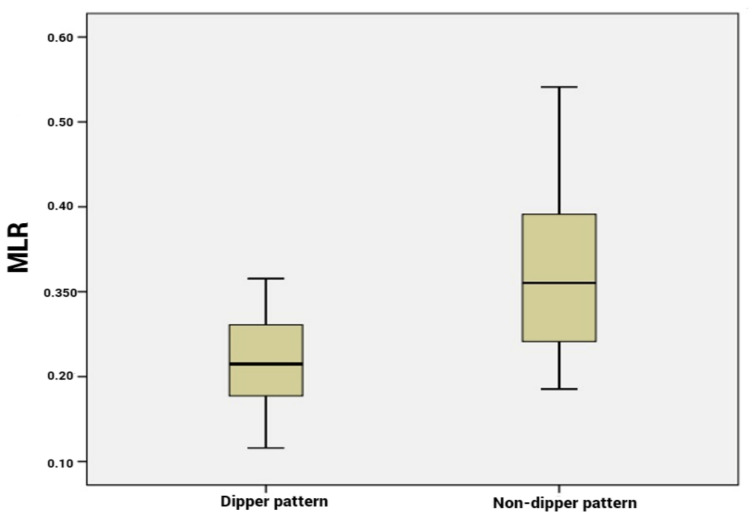
Monocyte to lymphocyte ratio levels according to dipper vs. non-dipper pattern.

Our analysis included 36 patients with dipping pattern and 44 patients with non-dipping pattern. Among the hematological parameters, the PLR, NLR and MLR were significantly higher in the non-dipping subgroup compared to the dipping subgroup (*p* < 0.001, Figure 1, Figure 2 and Figure 3). CRP and ESR were also higher in patients with non-dipper pattern; however, the difference was not statistically significant.

All patients were under lipid-lowering and antiplatelet therapy. One third of patients (34%) were under nitrate treatment. All patients used previous prescribed antihypertensive medication during ABPM. Angiotensin-converting enzyme inhibitor (ACEi)/ angiotensin receptor blocker (ARB), beta-blockers and thiazide-like diuretics were most frequent and their use was balanced between the two subgroups. Only 12% of patients were treated with central alpha antagonists. Calcium antagonist use was significantly more frequent in patients with a dipper circadian profile, compared to non-dippers (*p =* 0.001). The prevalence of monotherapy and dual antihypertensive therapy was slightly increased in the non-dipper pattern subgroup, but the difference between groups did not reach statistical significance. Details are shown in Table 2.

In a logistic multivariate model, PLR, MLR and calcium antagonist use remained significant predictors of non-dipper pattern (Table 3). Although NLR and lymphocyte count were significant predictors of non-dipper pattern in univariate analysis, their statistical significance was lost after inclusion in the multivariable regression model. 

## 4. Discussion

The primary result of this analysis was that MLR and PLR were elevated in non-dippers compared to dippers in patients with HTN and associated CAD. Both ESC and AHA HTN guidelines promote a more extensive use of ABPM in hypertensive patients in order to assess their circadian BP pattern. A non-dipping BP pattern is associated with elevated risk of atherosclerotic events and HTN-mediated target organ damage, accelerated CAD progression and an increased likelihood of obstructive sleep apnea [47,48,49]. Previous studies have documented increased inflammatory markers in both hypertensive and normotensive patients with a non-dipping BP profile [17]. Atherosclerosis is a dynamic low-grade inflammatory process [50,51,52,53] and routine inflammatory biomarkers are used for both acute and long-term cardiovascular risk stratification in CAD patients [10,13,54]. Neutrophils and monocytes are innate immune cells that are involved in the initiation and later activation of adaptive immunity, which plays a pivotal role in the pathology of hypertension and vascular injury [55]. While genetic studies have demonstrated that monocyte/macrophages are essential for angiotensin II-induced BP elevation and subsequent vascular dysfunction, neutrophils seem to play a more indirect role, promoting cardiovascular injury by activation of B and T lymphocytes [55]. In this context, the current study showed that the relationship between two inexpensive, routinely used inflammatory biomarkers and a non-dipper circadian BP pattern is maintained in the context of stable CAD association.

Endothelial dysfunction, a cause and effect of HTN, is preceded by alterations in the expression of cytokines and of endothelial cell receptors, as well as dysregulation in vascular smooth muscle, platelet and monocyte function [56]. Lymphocyte to monocyte ratio (LMR) was negatively associated with the prevalence of HBP in a recent cross-sectional study [57]. MLR was also a mortality predictor in COVID-19 patients [36]. Furthermore, MLR and PLR were associated with coronary artery ectasia severity in CAD patients [37]. More importantly, it was recently postulated that LMR is a novel marker for BP variability and HTN-mediated target organ damage in primary and secondary HTN in children [58]. NLR reflects vascular parietal inflammation with cardiovascular prognostic implications. NLR is a predictor of cardiovascular events and mortality and was associated with the extent of coronary atherosclerotic lesions in patients with stable CAD [51,52]. NLR levels are significantly correlated with an increased risk of developing hypertension [59,60] and is increased up to 72% in non-dipper hypertensive patients compared to dippers [41]. Kılıçaslan et al. showed that NLR is correlated with BP variability in both hypertensive and normotensive patients and suggested that elevated NLR is a predictor of increased HTN-mediated adverse cardiovascular events [61]. In a recent retrospective cohort, NLR and PLR were proposed as easily accessible markers of a non-dipper circadian profile in hypertensive patients [42]. Moreover, NLR was also associated with a reverse dipper pattern and exhibited a negative correlation with the decline rate of nocturnal systolic BP and diastolic BP [61]. Another study showed that NLR is elevated in subjects with resistant HTN compared to patients with controlled HTN [62].

Increased platelet activation plays a major role in initiation and progression of atherosclerotic lesions, and is associated with an elevated risk of plaque thrombosis. As such, PLR has been studied as a promising dual marker that reflects both inflammatory status and the extent of atherosclerosis [28,63]. Some studies showed that PLR is elevated in subjects with a non-dipping circadian profile [42,64,65,66]. Furthermore, PLR, but not NLR, was demonstrated to be an independent predictor of non-dipper circadian profile in a previous cohort of 166 hypertensive patients [41]. In another cross-sectional study, normotensive non-dipper patients had elevated PLR, similar to that of dipper hypertensive individuals, both higher than in dipper normotensive controls [67].

Although elevated CRP values have been previously associated with resistant and non-dipping essential hypertension [68], in our study the values of CRP and ESR were similar in patients with dipping and non-dipping circadian profile. High-sensitive C-reactive protein and carotid artery intima–media thickness are more sensitive short- and long-term prognostic cardiovascular biomarkers, but are too expensive to be routinely used in clinical practice [69].

Some classes of antihypertensive drugs (especially ACE inhibitors, ARB, b-blockers, and, to a lesser extent, calcium channel blockers [70]) possess anti-inflammatory effects shown by their ability to lower CRP levels. Several studies have focused on the “anti-inflammatory capacity” of the beforementioned antihypertensive classes. A study conducted by Fulop and colleagues showed that renin–angiotensin–aldosterone system inhibitors were more effective in reducing CRP levels than other antihypertensive drugs [71]. Nebivolol, a selective ß1-blocker, was studied by Fici et al. in a double-blind randomized trial. The authors reported that nebivolol not only decreases blood pressure values, but also modulates vascular microinflammation amelioration and reduces NLR in a manner independent and different from metoprolol [72]. Karaman et al. reported that valsartan (ARB) and amlodipine (calcium antagonists—dihydropyridines) were efficient in reducing NLR after 12 weeks of treatment in patients with newly diagnosed HTN [73]. A fixed dose combination of valsartan and amlodipine administered in a non-dipper hypertensive cohort, and compared to the same two antihypertensive drugs administered separately, showed a significant improvement in circadian blood pressure variation pattern in the polypill subgroup [74]. Besides careful medication selection, nighttime dosing of long-acting antihypertensive preparations demonstrated similar effects on nocturnal BP reduction and dipping rhythm restoration [75]. Despite emerging studies, the clinical impact of the anti-inflammatory properties of different classes of antihypertensive drugs is still insufficiently understood. Beyond medication, modification of traditional risk factors (BMI, smoking, and sedentary lifestyle) that affect inflammation becomes more of an issue in not only the control of HTN but also in decreasing comorbidities (CAD) and total cardiovascular risk.

Obesity, diabetes and the related autonomic dysfunction are associated with a higher prevalence of non-dipping status. Furthermore, an impaired glucose metabolism also favors systemic inflammation, explaining why NLR and PLR were previously demonstrated to be useful predictors of prediabetes and diabetes mellitus [5,76]. However, both BMI and the prevalence of diabetes had similar values in the two analyzed subgroups.

In our study, both NLR, MLR and PLR values were found to be higher in patients with a non-dipper pattern. On the other hand, CRP did not significantly vary between subgroups. ACEi/ARBs were the most frequently prescribed antihypertensive classes, and had a similar distribution between our two subgroups. However, calcium antagonist use was significantly lower in the non-dipper HTN.

To summarize, NLR, MLR and PLR are simple, readily available, inexpensive, and noninvasive parameters, which lately emerged as potent inflammatory and oxidative stress biomarkers. Because NLR, MLR and PLR are ratios, they are less prone to bias/variations with dehydration, over-hydration and blood specimen handling than other individual blood parameters taken separately [41,55]. Although current hypertension guidelines recommend an increased use of ABPM, the investigation has its own limitations [77]: nighttime discomfort, patient reluctance, potential movement artifacts, as well as relatively limited availability of the device in general practice. On the other hand, NLR, MLR and PLR can be obtained from a simple blood count, which is routinely performed in all medical and surgical specialties. As such, the value of these inflammatory biomarkers could provide an additional argument for ABPM referral.

To the best of our knowledge, our study is the first to analyze NLR, MLR and PLR levels in hypertensive patients with stable CAD. As inflammation plays a pivotal role in both hypertension and atherosclerosis, previous studies that analyzed the relationship between NLR, MLR, PLR and circadian BP variation excluded patients with CAD. Further studies should compare inflammatory biomarkers in hypertensive CAD patients with at least two different control groups: normotensive CAD patients and hypertensive patients without CAD. In our study MLR and PLR values, but not NLR values, were independent predictors for BP variability.

The present study has some limitations. Principally, it is a single-center retrospective analysis that included a limited number of subjects and lacked the aforementioned control groups. Other important limitations included the use of a single determination of NLR, MLR and PLR and the lack of consideration for hsCRP, carotid intima–media thickness and other inflammatory biomarkers. Furthermore, some antihypertensive drugs possess anti-inflammatory properties and could therefore influence CRP, MLR and PLR. Although the use of different antihypertensive classes was generally balanced between subgroups, heterogeneity remained regarding the dose and the particular agent used in each patient, as well as regarding BP optimal control. For this reason, we chose not to include hemodynamic data in our study and to limit our statistical analysis to dipping versus non-dipping pattern. Although all patients tested negative for COVID-19 upon hospital admission, we did not perform COVID-19 antibody tests to accurately exclude any prior COVID-19 infection.

## 5. Conclusions

Calcium antagonist use is associated with a higher likelihood of obtaining a physiological, dipper BP values pattern. PLR and MLR are significantly elevated in hypertensive stable CAD patients with a non-dipper BP pattern. These inexpensive and readily available laboratory parameters could prove valuable in the risk stratification of hypertensive CAD patients.

## Figures and Tables

**Table 1:** Characteristics of the two subgroups according to dipping status.

Parameters	All Patients (*n* = 80)	Dipper Pattern (*n* = 36)	Non-Dipper Pattern (*n* = 44)	*p* Value *
Age (years) ^×^	55.51 ± 11.83	53.28 ± 11.39	57.34 ± 12	0.12
Males, *n* (%) ^□^	57 (71.3)	27 (75)	30 (68.1)	0.42
Grade 1 HTN, *n* (%) ^□^	14 (17.5)	7 (19.4)	9 (20.4)	0.59
Grade 2 HTN, *n* (%) ^□^	23 (28.75)	11 (30.5)	11 (25)	0.98
Grade 3 HTN, *n* (%) ^□^	43 (53.75)	18 (50.1)	24 (54.5)	0.24
NLR ^†^	1.75 (1.39–2.58)	2 (1–2)	2 (2–3)	<0.001
PLR ^†^	147 (116–177)	116 (102–145)	175 (144–215)	<0.001
MLR ^†^	0.26 (0.2–0.32)	0.21 (0.17–0.26)	0.31 (0.23–0.39)	<0.001
Platelet count, ×10^3^/μL ^†^	243 (195–284)	237 (211–265)	252 (221–305)	0.08
WBC count, ×10^3^/μL ^†^	5.5 (4.7–7.1)	5.95 (5–7.1)	5.1 (4.55–7.25)	0.33
Neutrophil count, ×10^3^/μL ^†^	3.15 (2.47–4)	3.35 (2.49–3.93)	3.05 (2.47–4.41)	0.71
Lymphocyte count, ×10^3^/μL ^†^	1.72 (1.44–1.99)	1.93 (1.63–2.49)	1.54 (1.16–1.74)	<0.001
Monocyte count, ×10^3^/μL ^†^	0.42 (0.35–0.53)	0.41 (0.34–0.49)	0.44 (0.35–0.56)	0.21
CRP (mg/L) ^†^	0.41 (0.24–1.04)	0.37 (0.19–0.89)	0.42 (0.14–0.6)	0.68
ESR (mm/h) ^†^	12 (6–23.5)	11 (4.24–25)	12.5 (7–22)	0.54
BMI (kg/m^2^) ^†^	28.7 (27.4–33)	30.53 (27.4–33.02)	28.85 (27.2–32.7)	0.46
Diabetes, *n* (%) ^□^	22 (27.5)	10 (27.8)	12 (27.3)	0.96
HbA1c (%) ^×^	6.92 (6.01–7.46)	6.99 (5.81–8.29)	6.79 (6.1–7.42)	0.96
LDL (mg/dL) ^×^	107.7 (73.2–137.6)	105.9 (81.1–136.4)	110.6 (70.85–146)	0.74
HDL (mg/dL) ^×^	45 (39.02–56)	42.05 (38.17–54.67)	46.85 (39.35–57.8)	0.41
HDL/LDL ^×^	0.44 (0.31–0.6)	0.43 (0.31–0.58)	0.45 (0.31–0.69)	0.65

NLR: neutrophil to lymphocyte ratio, PLR: platelet to lymphocyte ratio, MLR: monocyte to lymphocyte ratio, CRP: C-reactive protein, ESR: erythrocyte sedimentation rate, BMI: body mass index, HbA1c: glycosylated hemoglobin, LDL: low density lipoprotein, HDL: high density lipoprotein, * Difference between dipper and non-dipper hypertension. Data are presented as follows: ^×^ Mean ± SD; ^□^ *n*, %; ^†^ Median (interquartile range).

**Table 2:** Hypertension treatment followed by patients at the time of ABPM.

Medication Class	All Patients (*n* = 80)	Dipper Pattern (*n* = 36)	Non-Dipper Pattern (*n* = 44)	*p* Value
Beta-blocker	65 (81.3)	28 (77.8)	37 (84.1)	0.56
ACE inhibitors/ARBs	63 (78.7)	30 (83.3)	33 (75)	0.62
Calcium antagonists (dihydropyridines)	27 (33.8)	23 (63.9)	4 (9.1)	0.001
Diuretics (thiazide-like)	67 (83.7)	27 (75)	40 (90.9)	0.62
Spironolactone	16 (20)	9 (25)	7 (15.9)	0.42
Central alpha antagonists	10 (12.5)	3 (8.3)	7 (15.9)	0.18
Drug treatment strategy				
Monotherapy	20 (25)	6 (16.7)	14 (31.8)	0.19
Dual combination	36 (45)	14 (38.8)	22 (50)	0.63
Triple combination	15 (18.7)	10 (27.7)	5 (11.3)	0.32
>3 drugs	9 (11.2)	6 (16.6)	3 (6.8)	0.25

All values are expressed as *n* (%). ACEi = angiotensin-converting enzyme inhibitor; ARBs = angiotensin receptor blocker.

**Table 3:** Multivariate regression analysis to predict non-dipper pattern.

Variables	Odds Ratio	95% Confidence Interval	*p*
Lymphocyte count, ×10^3^/μL	1.002	0.999–1.005	0.18
NLR	2333	0.439–12.392	0.32
PLR	1.071	1.024–1.120	0.002
MLR	6.64 × 10^6^	9.0645–4.86 × 10^12^	0.022
Calcium antagonists (dihydropyridines)	0.03	0.003–0.319	0.003

NLR: neutrophil to lymphocyte ratio, PLR: platelet to lymphocyte ratio, MLR: monocyte to lymphocyte ratio.

## Data Availability

Data are available from the corresponding author upon request.
